# An mCARE study on patterns of risk and resilience for children with ASD in Bangladesh

**DOI:** 10.1038/s41598-021-00793-7

**Published:** 2021-11-01

**Authors:** Masud Rabbani, Munirul M. Haque, Dipranjan Das Dipal, Md Ishrak Islam Zarif, Anik Iqbal, Amy Schwichtenberg, Naveen Bansal, Tanjir Rashid Soron, Syed Ishtiaque Ahmed, Sheikh Iqbal Ahamed

**Affiliations:** 1 Ubicomp Lab, Department of Computer Science, Marquette University, Milwaukee, WI 53233 USA; 2 R.B. Annis School of Engineering, University of Indianapolis, Indianapolis, IN 46227 USA; 3 College of Health and Human Sciences, Purdue University, West Lafayette, IN USA; 4 Department of Mathematical and Statistical Sciences, Marquette University, Milwaukee, WI USA; 5 Telepsychiatry Research and Innovation Network Ltd, Dhaka, 1215 Bangladesh; 6 Department of Computer Science, University of Toronto, Toronto, ON M5S 2E4 Canada

**Keywords:** Human behaviour, Risk factors

## Abstract

Community-wide lockdowns in response to COVID-19 influenced many families, but the developmental cascade for children with autism spectrum disorder (ASD) may be especially detrimental. Our objective was to evaluate behavioral patterns of risk and resilience for children with ASD across parent-report assessments before (from November 2019 to February 2020), during (March 2020 to May 2020), and after (June 2020 to November 2020) an extended COVID-19 lockdown. In 2020, our study *Mobile-based care for children with ASD using remote experience sampling method *(*mCARE*) was inactive data collection before COVID-19 emerged as a health crisis in Bangladesh. Here we deployed “Cohort Studies”, where we had in total 300 children with ASD (150 test group and 150 control group) to collect behavioral data. Our data collection continued through an extended COVID-19 lockdown and captured parent reports of 30 different behavioral parameters (e.g., self-injurious behaviors, aggression, sleep problems, daily living skills, and communication) across 150 children with ASD (test group). Based on the children’s condition, 4–6 behavioral parameters were assessed through the study. A total of 56,290 behavioral data points was collected (an average of 152.19 per week) from parent cell phones using the mCARE platform. Children and their families were exposed to an extended COVID-19 lockdown. The main outcomes used for this study were generated from parent reports child behaviors within the mCARE platform. Behaviors included of child social skills, communication use, problematic behaviors, sensory sensitivities, daily living, and play. COVID-19 lockdowns for children with autism and their families are not universally negative but supports in the areas of “Problematic Behavior” could serve to mitigate future risk.

## Introduction

In the first quarter of 2021, the COVID-19 disease caused by the coronavirus (SARS-CoV-2) continues to demonstrate worldwide super spreading power^1–5^. Though it was first identified and reported in central China in December 2019, it is now a global concern in all sectors of human life after its devastating infection rate and deadly threat^6,7^. To mitigate the infection rate, during the initial period of COVID-19, many countries in Europe, Asia, and America were under lockdown^8,9^. According to reports from UNSDG^10,11^ and UNESCO^12,13^, more than 188 countries closed schools starting in April of 2020. Social isolation and loneliness can negatively impact children^10,14–16^, especially those with an Autism Spectrum Disorder (ASD)^17–20^. Autism Spectrum Disorder (ASD) is a heterogeneous neurodevelopmental disorder^21^, which was first described as children’s behavioral disorder by Kanner in 1943^22^. Now, it is a global problem in the world^23^. Quarantine and lockdown can directly hamper the neurodevelopmental and behavioral treatment for children with ASD^24,25^. It can also increase parental and caregiver stress^26^. Today, most countries have relaxed their lockdown policies^27^; however, many schools, including specialized schools, remain closed^12^. The long period of school shutdown prevented children with ASD from getting therapy for behavioral and developmental improvement^18,24,25^. The lockdown due to COVID-19 has also revealed the potential gap in the mental health services of children with ASD during emergencies^28^. This study reveals the impact of the lockdown on behavioral development in Bangladesh for children with ASD.

In Bangladesh, the COVID-19 impacts on children’s mental health, especially by depression, anxiety, and sleeping disorder. Approximately 19.3% of Bangladeshi children were disturbed moderately, and about 7.2% were severely suffered by COVID-19^29^. This children’s mental disturbance is also linked to parents’ stress and abnormal behavior, especially for the children with ASD. Children with ASD were at risk for delays during the lockdown period^8^. According to the DSM-5^30^, new lifestyle changes can create difficulties in their behavioral development. Several studies^1,8,10,18,31–33^ document that the COVID-19 confinement impacts the psychological wellbeing of children with ASD. Most studies were based on surveys or focus-group interviews from parents. But behavioral changes in a child with ASD can be a long process, so to get a better picture of long-term impacts, prospective longitudinal studies are needed on children with ASD. Our aim in this study was to document the impact of the COVID-19 lockdown on specific behavioral parameters of children with ASD.

Our work is the first prospective longitudinal study to examine the COVID-19 lockdown effect on the specific behavioral parameter in ASD within a Low-Middle-Income-Countries like Bangladesh. This study is based on the mCARE (*Mobile-based care for children with ASD*) project^34,35^, which monitored 150 children with autism, capturing behavioral data for one year (from November 2019 to November 2020). In Bangladesh, the first lockdown took place from March 2020 to May 2020^36^. The timeframe of our study provided us with pre-lockdown (from November 2019 to February 2020), lockdown (March 2020 to May 2020), and post-lock down (June 2020 to November 2020) participant behavioral data. Using the mCARE tools over the course of the pandemic restrictions, health care professionals could take proper care and employ evidence-based decision-making remotely^34^. This study aims to evaluate parent-report behavioral patterns of risk and resilience for a group of children with ASD before, during, and after an extended COVID-19 lockdown. The main aim of mCARE was to monitor the behavioral and milestone parameter of the children with ASD by remote sampling method using the mobile app. The project was held in Bangladesh for a year, and in this time period, the COVID-19 pandemic was broken out and, several countries were locked down including, Bangladesh. In this study, we used the mCARE test group children’s behavioral data to find the research question that “How lockdown due to COVID-19 impacts on children with ASD?”. We hypothesis that “The lockdown due to COVID-19 has more negative behavioral impact than positive impact on children with ASD”. However, it has some positive impacts on children with ASD.

## Methods

In this study, we evaluate the behavioral changes (patterns of risk and resilience) for 150 children with ASD in Bangladesh. We used the mCARE^34,35^ data and divided the data into three time-based segments (pre-lockdown, lockdown, and post-lockdown data) based on the COVID-19 lockdown period in Bangladesh. After that, we analyzed the data and compared the behavioral patterns of risk and resilience.

### mCARE

In this project, a total of 300 children with ASD were recruited from four major centers in Bangladesh. We have selected these four centers, there are the major Autism Development centers in Bangladesh, and we recruited these children by proper consent from these centers, and we got 95% of positive responses from the parents of children with ASD. Besides, we also took the IRB from Marquette University Institutional Review Board on July 9, 2020, with the protocol number HR-1803022959 for recruiting these children. Then we have divided two groups: (a) test group: 150 children with ASD are in this group who are regularly monitored by the mCARE system. (b) control group: 150 children with ASD are in this group who are not monitored regularly by the mCARE system, but they were in the system to compare with the test group children. We selected the children for these groups randomly from our cohort. In this 300 cohort, we selected children aged from 2 to 9, and where 79.7% were male, and 20.3% were female, 61.9% were active smartphone users (APP users), and the rest of 38.1% used non-smartphone users (SMS users). We observed that parents were interested in mCARE: APP using, as they feel easier use in mCARE: APP. And we also observed that mCARE: APP users responded higher and more accurately than mCARE: SMS users. We have recruited all demographic children with ASD, such as single parents who have only fathers or mothers. Recruitment had been done by voluntary participation by the legal guardians and will be conducted through the care practitioners. In the recruiting process, we have faced the challenge of training the parents, but by focus group discussion, we overcame the problem. We have analyzed the practitioner satisfaction based on paired t-test^37^. In this study, we have used the “test group” children data to evaluate the behavioral changes in the COVID-19 period.

### Research design

In this study, we analyzed the data from the mCARE project, collected from November 2019 to November 2020. mCARE, an innovative longitudinal monitoring digital platform, was funded through a grant from the National Institutes of Health (NIH)^38^ and implemented in Bangladesh. The mCARE tools included an mCARE: APP (for smartphone-based service) and mCARE: SMS (a short message service) for families with a cell phone without a ‘smart’ interface. The mCARE project was run for one year with different activities. Our mCARE project included data from the pre-lockdown, lockdown, and post-lock down periods in Bangladesh^36^. In this study, we segmented these data into three sections (pre-lockdown, lockdown, and post-lockdown data) and analyzed the behavioral impact of the children with ASD by these groups of data.

### Participants

In this study, we have analyzed behavioral data from 150 ASD children enrolled in the “test group” of the mCARE project. In the mCARE project, we enrolled 300 children with ASD from Bangladesh between 2 and 9 years of age with approval from Marquette University’s Institutional Review Board (Protocol number HR-18030222959). We took the informed consent from the parents and/or legal guardians of all subjects (as all subjects were under 18 years) in mCARE to analyze their data for research purposes. Children with ASD were recruited from the four major institutes of Bangladesh in two geographical locations—Dhaka and Chittagong. Typically, families in Bangladesh with low and high socioeconomic resources receive treatment from the public and private organizations, respectively. To include participants from all socioeconomic classes, we included two government organizations (NIMH^39^ and IPNA^40^) and two private organizations (AWF^41^ and Nishpap^42^). Table 1 shows the patient distribution (only test group) among the four centers. We have incorporated diversity in terms of age, sex, ASD severity, and family socioeconomic resources. From NIMH and IPNA, we recruited 100 caregivers of ASD kids from each. The participants have been divided into two groups—mCARE-APP (50) and mCARE-SMS (50). Each group was further divided equally into test (25) and control (25) groups. Similarly, from Nishpap and AWF, 50 participants have been chosen from each school divided into a test group (25) and a control group (25). The power analysis yields that the sample size of 300 is sufficient to discover a difference among mCARE-SMS, mCARE-APP, and the control groups with the power of 0.97 at α = 0.05 and the medium effect size. The power analysis was performed using GPower 3.1 based on the ANOVA with all covariates, including demographic variables, care facilities, and care practitioners. (detailed description of the recruitment process in “mCARE” section).

**Table 1:** The patient distribution among the four centers.

Serial	Center name	Patients distribution
		Test group
1	The National Institute of Mental Health (NIMH)^39^	50
2	The Institute of Pediatric Neuro-disorder and autism (IPNA)^40^	50
3	Autism Welfare Foundation (AWF)^41^	25
4	Nishpap Autism Foundation^42^	25
Total		150

The sociodemographic information of this study is shown in Table 2. In Table 2, the total number of participants is 300 (both the test and control groups). These groups were divided into two categories (mCARE: APP and mCARE: SMS) based on the mCARE tool application. In this work, we only use the “Test Group” behavioral data to analyze the impact of COVID-19 on children with ASD in Bangladesh.

**Table 2:** Participant’s sociodemographic information.

Features		mCARE (%)	mCARE: APP		mCARE: SMS	
			Test group (%)	Control group (%)	Test group (%)	Control group (%)
**Demographics of children**						
Age	2–6	25.9	7.5	6.6	4.6	7.2
	6–9	74.1	24.9	23.0	13.1	13.1
Sex	Male	79.7	27.2	21.6	14.4	16.4
	Female	20.3	5.2	7.9	3.3	3.9
Education	Never went to school	28.9	6.6	8.2	5.6	8.5
	Went to usual academic school but failed to continue study	13.1	3.6	3.0	3.6	3.0
	Went to specialized school but failed to continue study	3.0	1.0	0.3	0.3	1.3
	Currently he/she is going to usual academic school	6.2	2.3	1.6	1.6	0.7
	Currently he/she is going to specialized academic school	48.9	19.0	16.4	6.6	6.9
**Demographics of primary caregivers**						
Education	Primary	19.7	3.3	4.4	5.6	6.4
	Secondary	20.5	5.7	6.4	4.1	4.3
	Higher secondary	59.8	23.5	18.7	8	9.7
Occupation	Housewife	81.7	25.6	22.6	15.1	18.4
	Service	13	4.9	5.2	1.6	1.3
	Others	5.3	2.0	1.6	1.0	0.7
Average family spending/month (thousand BDT)	< 15	13.4	3.9	1.6	3.3	4.6
	15–30	29.5	8.5	8.2	5.9	6.9
	30–50	22.3	5.2	7.5	4.9	4.6
	> 50	34.8	14.8	12.1	3.6	4.3
Family type	Nuclear	75.7	24.9	22.6	12.1	16.1
	Extended	24.3	7.5	6.9	5.6	4.3
Geographic location	Urban	76.7	27.2	24.3	12.1	13.1
	Semi-urban	12.1	3.3	3.0	2.6	3.3
	Rural	11.1	2.0	2.3	3.0	3.9

### Instruments

We deployed the two mCARE tools to collect behavioral parameters based on family phone use/access across the four recruitment locations. We provided the mCARE tools to the primary caregivers (mostly the biological parents of children with ASD).

We assessed 30 behavioral parameters. Initially, the study’s care practitioners (clinical coordinators of the four centers) set the behavioral parameters (4–6 parameters for each child) for the children with ASD based on their initial screening and intervention. So about 13.33–20% of behavioral parameters were used to assess every child in this study. After the initial setup, the parents/caregivers began reporting their children's behavioral data based on their child's current behaviors/progress twice a week. A solid data collection plan with proper consent was provided at the begging of the study. All the data was saved in mCARE: DMP (Data Management Platform).

### Role of the clinical coordinators and caregivers

In mCARE, there were 16 clinical coordinators had been recruited for four centers, and for each child with ASD, there was one particular primary caregiver. In this study, the clinical coordinator and caregiver have played a vital role in fulfilling the study’s goal. The clinical coordinators are responsible to (i) monitor and utilize the data submitted (in mCARE-DMP) by the caregivers in their treatment process, (ii) follow-up with the primary caregiver if data collection were interrupted, (iii) support the caregivers in any abnormal situation like lockdown in COVID-19, (iv) update the behavioral and milestone parameters of children (with ASD) in the system (mCARE: DMP) based on their condition, (v) communicate with the caregiver, (vi) conduct focus group session with the caregiver, and (vii) report the critical findings to the research team. As the mCARE is a self-reported study for longitudinal monitoring of children with ASD, the primary caregivers (parents) are the key person of the study. Their main role is to (i) report their children's progress based on their condition through mCARE: APP or mCARE: SMS, (ii) take-care their children according to the clinical coordinators’ suggestions and ASD knowledge getting from the mCARE, and (iii) contact with the clinical coordinator if there any emergency help needed. The family supports and dwelling environment are two important factors in this study. We observed that those children who belong to fewer siblings and higher income-educated families could get better family care; that reflects on their better improvement level. Similarly, we also observed better improvement in our “test group” children (with ASD) who had better living places, especially in urban rather than rural and congested slums. In the lockdown period due to COVID-19, the children living in congested slums or apartments were more containment than other children who lived in space areas or homes.

### Data collection rate

**Figure 1:**
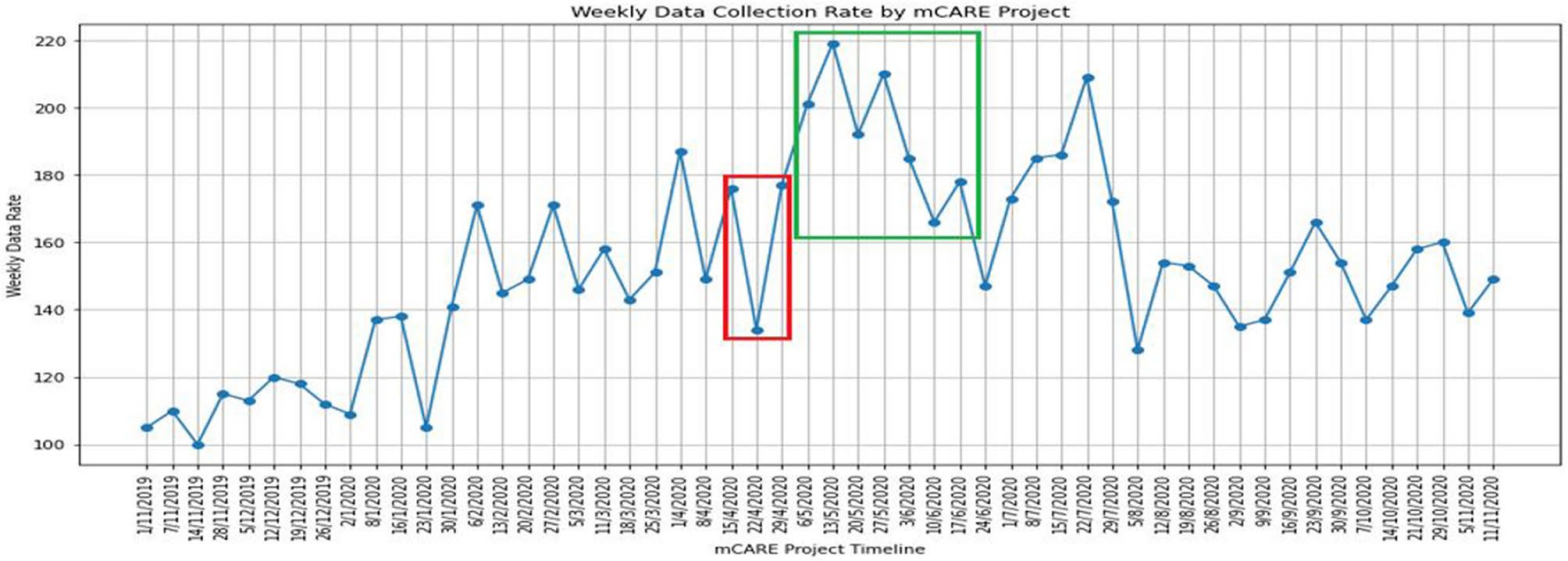
mCARE data collection rate (weekly).

The average data collection rate of mCARE was around 152.19 per week from the four centers. Figure 1 shows the data collection rate throughout the mCARE project timeline. In the data collection phase, we faced several difficulties during the lockdown period.

We observed a low data collection rate (shown in the red box in Fig. 1) during the Bangladesh lockdown time. Several reasons were discovered:

I. Several patients returned to their hometowns, mostly in rural areas, where the network connection was unreliable.
II. In some cases, for job purposes, the father had to stay separate from the family, taking the single-family mobile phone with him.
III. Many parents lost their jobs or had limited income, so they faced difficulties submitting data. We provided all participants monthly incentives throughout the study.
IV. Family members and caregivers experienced COVID-19-like symptoms and went into isolation in a separate room in the home. This hampered the family from giving regular data feedback.
V. Caregivers had mobile phone issues, such as the mobile phone they used to send data was broken or had no balance, and they couldn't repair it or recharge it in time due to the lockdown situation.
VI. As the schools were closed due to the lockdown, some of the patients changed schools, and some went to their hometowns permanently.

Our clinical coordinators handled individual problems, talked with participants on their cell phones, and offered assistance and motivation to find solutions for data reporting. As many of the families were in financial crisis in the COVID-19 lockdown period for no job, business, or leaving their home and shifting to the village, our clinical coordinator found these families and helped them financially from the mCARE project fund. It was important to collect proper children’s data from the parents to get the impact of COVID-19 on children’s mental health. As individual problems were handled and resolved, data collection rates rose among all participants (shown in the green box in Fig. 1).

### Statistical analysis

The goal of this study was to determine how lockdown impacted children with ASD based on parental behavioral reports. We considered 30 behavioral parameters (both positive and negative based on the behavioral type), and based on the psychiatrist’s expertise, we categorized them into four domains (Communication, Social Interaction, Problematic Behavior, and Sensory Sensitivities) (Details in Table 3). Parents were asked to provide the rated severity (0 to 10) of each behavior. Parents provided this value using the mCARE: APP or mCARE: SMS). The evaluation window was considered for a year. We divided this window into three different phases (pre-lockdown, lockdown, and post-lockdown) to differentiate the lockdown impact on the children with ASD compared to normal time. Simple descriptive statistics (mean^43^ and mode^43^) were utilized to calculate behavior severity for three different phases. We also calculated the 95% Confidence Interval (CI) for the validation of our results. We used the “Z” value (1.96 for 95% CI)^44^ for calculating the 95% CI using the following formula:

$$\overline{X}\pm Z\frac{s}{\surd n },$$

where $\overline{X }$ is the mean; Z is 1.96, chosen from the Z-value table^44^; S is the standard deviation; n is the sample number in average.

**Table 3:** Comparison behavioral changes of ASD children between the lockdown period and normal period (calculated by statistical mean with 95% CI).

Domain	Parameters	Behavioural type	Before lock down period (pre-lockdown) (Nov-2019 to Feb-2020)				During lock down period (lockdown) (March-2020 to May-2020)				After lock down period (post-lockdown) (June-2020 to Nov-2020)				Impact by the lock down
		Negative/positive	N	Avg. Behavioural Changes Level	Lower bound of CI	Upper bound of CI	N	Avg. behavioural changes level	Lower bound of CI	Upper bound of CI	N	Avg. Behavioural changes level	Lower Bound of CI	Upper bound of CI	Negative/positive/no
Communication	Word repetition	Negative	121	7.4	7.2	7.6	92	6.8	6.6	7.01	169	6.7	6.5	6.9	No
	Use of meaningless words	Negative	121	7.03	6.9	7.2	92	6.4	6.2	6.5	169	5.6	5.5	5.8	No
	Misuse of pronouns (such as “I” instead of “you”)	Negative	108	5.9	5.5	6.3	91	5.2	4.9	5.6	157	4.8	4.6	5.08	No
	Use of unnatural sounds (e.g. high pitch squeal)	Negative	120	6.6	6.3	6.9	92	5.9	5.6	6.15	167	5.6	5.4	5.8	No
	Response to name	Positive	120	5.8	5.6	5.9	92	5.4	5.3	5.5	169	4.8	4.7	4.9	Negative
	Delay in response (social/motor/interactive)	Negative	121	5.9	5.6	6.06	92	5.4	5.2	5.7	168	4.5	4.3	4.7	No
	Avoids eye contact	Negative	121	5.4	5.1	5.6	92	5.5	5.3	5.7	168	5.3	5.2	5.5	Negative
	Understands personal care routine	Positive	121	5.9	5.7	6.2	92	5.2	5.02	5.4	169	4.8	4.6	4.9	Negative
	Fails to express basic needs (e.g. hunger)	Negative	111	4.8	4.5	5.2	89	4.1	3.8	4.5	160	4.1	3.8	4.4	Positive
Social interaction	Can s/he start social interactions?	Positive	121	5.9	5.7	6.2	92	5.2	4.9	5.5	169	5.5	5.3	5.7	Negative
	Can s/he maintain social interactions?	Positive	114	5.7	5.4	6.0	92	4.7	4.5	5.04	164	4.8	4.8	4.9	Negative
	Use of social smile	Positive	76	6.3	5.7	6.9	83	5.2	4.7	5.8	134	3.5	3.04	4.04	Negative
Problematic behavior	Mood swings	Negative	118	8.9	8.8	9.04	92	7.9	7.7	8.2	169	6.5	6.3	6.8	No
	Self-injurious behavior (frequency)	Negative	118	6.07	5.6	6.5	91	2.9	2.6	3.2	164	3.8	3.4	4.08	Positive
	Self-injurious behavior (intensity)	Negative	77	6.3	5.5	7.1	62	5.1	4.02	6.2	99	5.2	4.4	6.09	Positive
	Aggressive behavior (frequency)	Negative	90	6.8	6.2	7.2	80	5.7	5.4	6.03	123	5.1	4.9	5.4	No
	Aggressive behavior (how often)	Negative	23	2.04	0.99	3.09	27	4.7	3.7	5.7	47	8.3	7.9	8.7	Negative
	Intense interest in objects/parts of objects	Positive	117	6.5	6.2	6.8	91	6.2	5.9	6.4	168	5.5	5.4	5.7	Negative
	Inflexible to change	Negative	91	6.2	5.8	6.6	77	5.2	4.8	5.6	148	6.0	5.7	6.4	Positive
	Repetitive activities (e.g., spinning objects)	Negative	121	7.02	6.8	7.2	92	6.2	6.02	6.3	169	5.9	5.8	6.04	No
	Hyperactive	Negative	121	7.1	7.0	7.5	92	7.3	7.1	7.5	169	6.6	6.5	6.8	Negative
	Lack of concentration	Negative	121	7.0	6.8	7.3	92	7.3	7.2	7.5	169	6.9	6.8	7.07	Negative
Sensory sensitivities	Difficulty tracking moving objects/people	Negative	116	6.4	6.03	6.7	91	6.02	5.7	6.3	163	4.9	4.6	5.2	No
	Participation in imaginative games	Positive	114	5.7	5.4	6.03	92	5.6	5.3	5.8	165	5.08	4.9	5.3	Negative
	Sleep problems	Negative	86	5.7	5.4	6.3	73	5.9	5.1	6.0	124	4.2	3.7	4.8	Negative
	Unusual sensitivity to light	Negative	59	3.6	2.9	4.2	70	2.2	1.7	2.6	119	2.2	1.9	2.4	Positive
	Sensitivity to pain	Negative	48	4.8	4.3	5.5	59	4.9	4.6	5.4	95	5.0	4.6	5.4	Negative
	Sensitivity to sound	Negative	68	7.1	6.6	7.7	77	5.8	5.3	6.4	163	4.6	4.3	4.9	No
	Aversion to smell	Negative	89	7.2	6.8	7.7	85	6.3	5.9	6.8	165	3.7	3.4	4.02	No
	Sensitivity to touch	Negative	45	4.7	3.9	5.6	63	3.5	2.9	4.08	92	3.3	2.8	3.8	No

In this study, we have followed our method in accordance with the relevant and standard guidelines and regulations.

### Patterns of risk and resilience

We have created Table 3 to show the patterns of risk and resilience on the behavioral parameter due to the COVID-19 lockdown. We analysed by both statistical mean (shown in Table 3) and mode (shown in Online Appendix Table S1) to determine the impact of the COVID-19 lockdown for children with ASD. We had a total of 56,290 behavioral data reports on the sum of all patterns for 150 families over one year (November 2019 to November 2020). We classified these data into three categories: before lockdown or pre-lockdown time (from November 2019 to February 2020), during lockdown (from March 2020 to May 2020), and after-lockdown or post-lockdown (from June 2020 to November 2020). We also categorized the 30 behavioral parameters into two types: positive parameters (7 out of 30) and negative parameters (23 out of 30) (Details in Table 3).

We calculated the month-wise behavioral severity rate for a particular behavioral parameter by taking the average (using the statistical mean) and most likely value (using the statistical mode). We calculated the pre-lockdown, lockdown, and post-lockdown for a particular behavioral parameter by averaging the month-wise behavioral severity rate. We calculated the impact table both for the statistical mean (shown in Table 3) and mode (shown in Online Appendix Table S1) with a 95% Confidence Interval (CI) to validate our result. From Table 3 and Online Appendix Table S1, we found the same impact on every behavioral parameter of the study participants.

In Table 3, we used the same concept to discover the positive and negative impact on a behavioral parameter due to lockdown. We also categorized our 30 behavioral parameters into four domains: (i) Communication, (ii) Social Interaction, (iii) Problematic Behavior, and (iv) Sensory Sensitivities. For a “Negative” behavioral parameter, if the lockdown period’s behavioral rate was higher than the pre-lockdown period’s data, then it created a “Negative” impact due to lockdown for that particular behavioral parameter. If the lockdown period’s behavioral rate was lower than both the pre-lockdown and post-lockdown period’s data, then it created a “Positive” impact. If the behavioral data was consistently decreased, then there was no impact of lockdown for that particular parameter. This rule is the opposite for calculating the impact on the “Positive” behavioral parameters.

## Results

**Figure 2:**
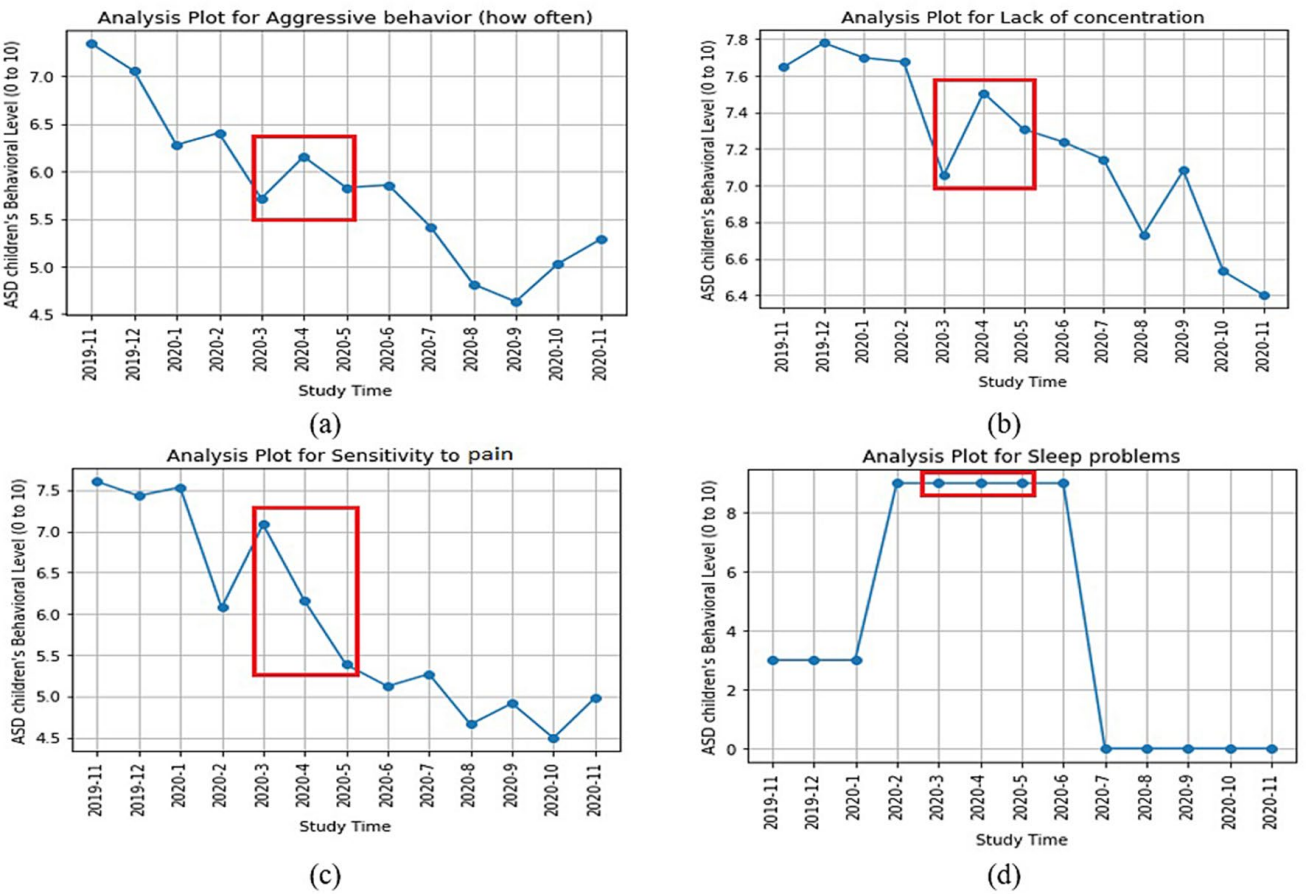
Graphical representation of the “negative impact” due to lockdown on children’s with ASD behavioral parameter: (**a**) aggressive behavioral (how often), (**b**) lack of concentration, (**c**) sensitivity to pain, and (**d**) sleep problem.

**Figure 3:**
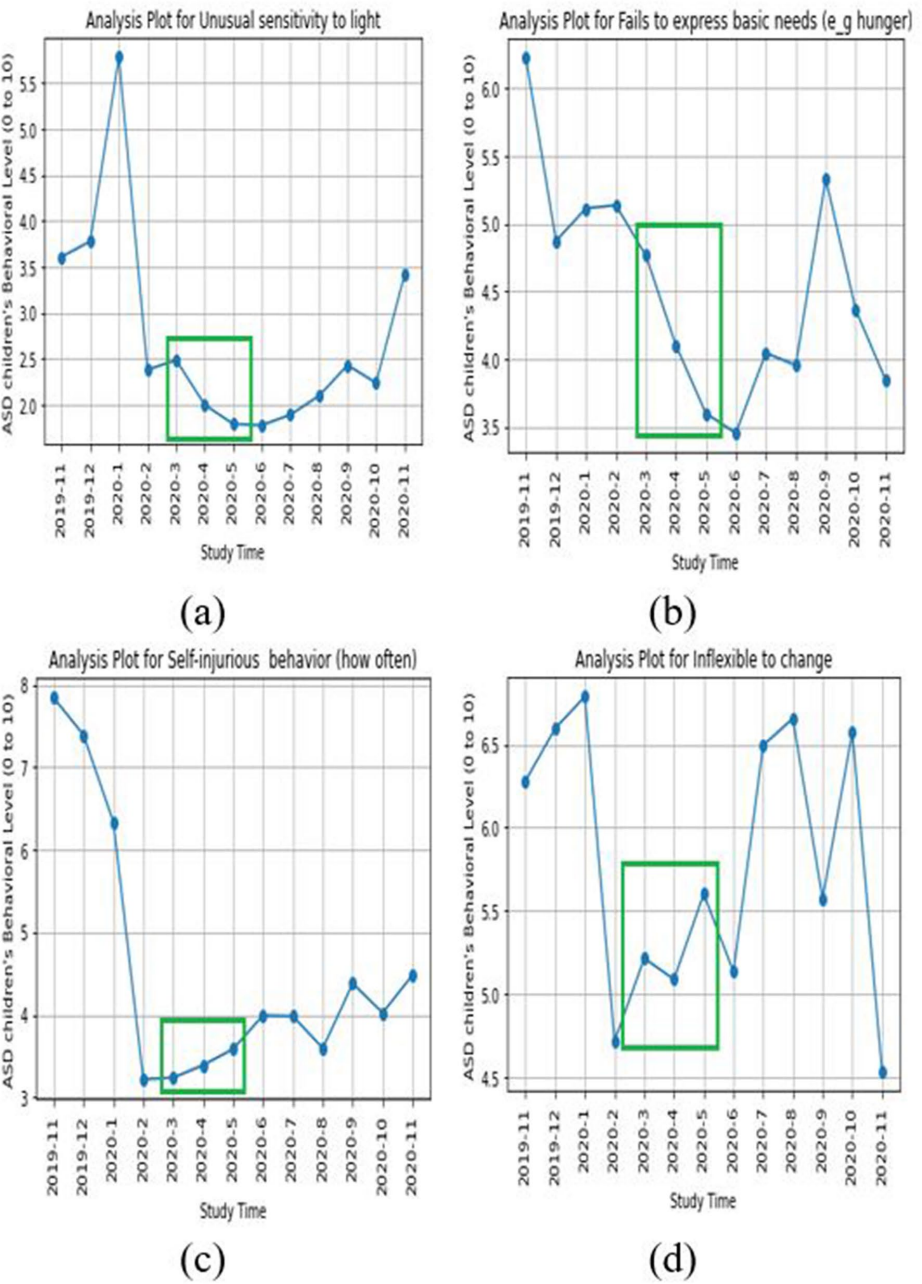
Graphical representation of the “positive change” due to lockdown on children’s with ASD behavioral parameter: (**a**) Unusual sensitivity to light, (**b**) Fails to express basic needs (e.g., hunger), (**c**) self-injurious behavior (how often), and (**d**) Inflexible to change.

Table 3 and Appendix Table S1 show the different levels of behavioral data in three different periods. Table 3 has been calculated by Statistical Mean, and Online Appendix Table S1 is calculated by Statistical Mode. Both tables use the same data but in different statistical models. We have used here 30 different behavioral parameters with the average value in a certain time period (Nov-2019 to Feb-2020, March-2020 to May-2020, and June-2020 to Nov-2020). We also classified these 30 behavioral parameters into four specific domains to understand better the COVID-19 impact on children with ASD. Initially, we also categorized the behavioral parameters by positive or negative, based on the behavioral type of the parameter, and it was also used to determine the COVID-19 impact on the children with ASD during the lockdown in Bangladesh. Overall from the two tables (Table 3, Online Appendix Table S1), we found the same result of impact rate due to lockdown. We found 13 “Negative” impacts out of 30 behavioral parameters, which is 43.33%, and 5 “Positive” impacts out of 30 behavioral parameters, which is 16.67%. The remaining 12 behavioral parameters had no impact (40%) due to the lockdown. Figure 2 shows the four negatively impacted behavioral parameters on children with ASD during the lockdown period. From Fig. 2a, we can see the aggressive behavior (how often) was increased in the lockdown period; similarly, the lack of concentration (Fig. 2b), the sensitivity of pain (Fig. 2c), and sleep problem (Fig. 2d) also increased on the lockdown time. According to our expert psychiatrists, this increased level of children’s behavior has been considered as a negative impact by the COVID-19 lockdown. Figure 3 shows the four positively impacted behavioral parameters on children with ASD during the lockdown period. From this figure, we can see the unusual sensitivity of light (Fig. 3a), basic needs like hunger (Fig. 3b), self-injurious behavior (how often) (Fig. 3c), and inflexible to changes (Fig. 3d) was decreased on the lockdown period, which indicates the positive impact of COVID-19 on the children with ASD. From the two tables (Table 3, Online Appendix Table S1), we can see there are 12 behavioral parameters (“No” in the “Impact by Lockdown” column) which has not changed during the lockdown period, and other five behavioral parameters (“Positive” in the “Impact by Lockdown” column) have improved on the lockdown time period in Bangladesh. This refers that COVID-19 lockdown has a resilience impact on the children (with ASD) behavior. On the other hand, the other 13 behavioral parameters (“Negative” in the “Impact by Lockdown” column) have a negative impact, which is surely linked to increasing the parents’ stress.

### Approval for human experiments

We took the IRB from Marquette University Institutional Review Board on July 9, 2020, with the protocol number HR-1803022959, and titled “MOBILE-BASED CARE FOR CHILDREN WITH AUTISM SPECTRUM DISORDER USING REMOTE EXPERIENCE SAMPLING METHOD (MCARE)” for recruiting a total of 316 subjects, of which we recruited 300. (Details description of participants in the “Methods” section).

## Discussion

### Important findings

We found a 43.33% negative impact of lockdown on the behavioral parameters; out of 30 behavioral parameters, 13 had a negative impact due to lockdown. One important finding from the result is, we got a “negative” impact for all seven positive behavioral parameters in our study. We conclude that lockdown had a more serious impact on the “positive behavioral parameter” than the “negative behavioral parameter”. Besides, among the four groups of 30 behavioral parameters, due to the lockdown of COVID-19, the test group children have been more suffering in the “Social Interaction” group. Table 3 found that three behavioral parameters out of 3 (100%) parameters have been negatively affected by the COVID-19 lockdown period. We have chosen and shown graphically (in Fig. 2) four “negative behavioral parameters” where we can observe the negative impact. In Fig. 2, we have marked the negative impact of the red box during the lockdown time.

Because of the assistance provided by mCARE and more care time given by the family member, we saw positive changes in study participants’ behavioral parameters even in the lockdown period. We have selected four behavioral parameters and represent the positive changes (marked by green box) graphically in Fig. 3.

### mCARE contribution in COVID-19 lockdown time

During the lockdown period due to COVID-19, the clinical coordinator could continue to give therapy to study participants based on the data from mCARE. For example, Table 4 shows the clinical professional's treatment process in the lockdown period based on the study participant’s condition. In this table, we categorize the parameter into four types: communication, daily living skills, motor skills, and socialization.

**Table 4:** Decision taken by clinical coordinator using mCARE in lockdown period.

Parameter types	Advise/decision/action taken by clinical coordinator in lockdown
Communication	To learn the Body parts by showing on the Mirror, advise to give picture-based storybooks
Daily living skills	Toilet training by scheduling and picture chat, Tooth brash by scheduling, use DTT method, through the home-based program, Follow the picture chat
Motor skills	Through verbally Modelling, try to group work with siblings, advised to draw a circle by using any circular thing like a ball
Socialization	Communicate with other family members, everyday walking on the field or roof or yard of the house

Based on the children with ASD behavioral data through mCARE: APP or mCARE: SMS, the clinical coordinator could make the proper evidence-based decision for each child with ASD during the lockdown.

Though several works^45,46^ in this relevant area to determine the mental health of the children with ASD in Bangladesh, in this study, we specifically identify the behavioral changes of the children with ASD in Bangladesh during the COVID-19 lockdown period. Compared to other studies, in this work, we have deployed our developed mCARE: APP and mCARE: SMS tools among our cohort. Besides that, our clinical coordinator also conducted the FGD to validate the cohort’s data and feedback.

### Strength and limitations

The strong cohort of 300 children with ASD (“Test Group” and “Control Group”) with their parent’s support and the value-sensitive design-based mobile application (mCARE: APP and mCARE: SMS) is the main strength of our study. As the behavioral development of children with ASD is a long-term process, we faced the challenge of keeping the parents’ motivation for providing the data regularly in this study. For this reason, we failed to maintain the same data collection rate (details in Fig. 1) from our study population over the study. In this study, we only considered the “test group” of our population with no “Gender-Specific” findings. And we have used our self-created 30 behavioral parameters, which had not been evaluated by standard instruments.

## Conclusions

In conclusion, this study indicates that the ongoing COVID-19 pandemic and the subsequent lockdown have undoubtedly produced a mental impact on young children with ASD and their surrounding caregiver personnel. In our research, we found the daily routine change, to be bound in the home for a long time, closed mental health centers for an extended period, the increase of difficulties and mental pressure of parents are the main critical factors underlying behavioral problems of children with ASD due to the COVID-19 outbreak. In the confinement period, children with ASD mainly suffered in their positive behavioral parameters like participating in-game, understanding their daily routine, intense interest in toys or dolls, and making social interaction or response. On the other hand, the effectiveness of in-home care and the potential use of online intervention tools like mCARE played a vital role in developing children with ASD at any time, both in normal or any pandemic time in the future.

## Supplementary Information

Supplementary Information.
