# Supplementary material for: An mCARE study on patterns of risk and resilience for children with ASD in Bangladesh
Source: Sci Rep. 2021 Nov 1;11:21342. doi: 10.1038/s41598-021-00793-7 (PMC8560902; doi:10.1038/s41598-021-00793-7)
Supplement: Supplementary file 1 — Supplementary Information. [file 41598_2021_793_MOESM1_ESM.docx]

**Appendix Table 1.**

**Appendix Table 1:** Comparison Behavioral Changes of ASD children between the lockdown period and normal period (Calculated by Statistical Mode with 95 % CI)

| **Domain** | **Parameters** | **Behavi- oural Type** | **Before Lock Down Period (Pre-Lockdown)**  **(Nov-2019 to Feb-2020)** | | | | **During Lock Down Period (Lockdown)**  **(March-2020 to May-2020)** | | | | **After Lock Down Period (Post-Lockdown)**  **(June-2020 to Nov-2020)** | | | | **Impact by the Lock Down** |
| --- | --- | --- | --- | --- | --- | --- | --- | --- | --- | --- | --- | --- | --- | --- | --- |
|  |  | **Negative / Positive** | **N** | **Avg. Behavi- oural Changes Level** | **Lower Bound of CI** | **Upper Bound of CI** | **N** | **Avg. Behavi- oural Changes Level** | **Lower Bound of CI** | **Upper Bound of CI** | **N** | **Avg. Behavi- oural Changes Level** | **Lower Bound of CI** | **Upper Bound of CI** | **Negative / Positive / No** |
| **Communication** | **Word repetition** | **Negative** | 202 | 7.8 | 7.5 | 8.2 | 161 | 7.8 | 7.4 | 8.09 | 325 | 7.01 | 6.7 | 7.3 | **No** |
|  | **Use of meaningless words** | **Negative** | 199 | 7.5 | 7.2 | 7.9 | 166 | 6.9 | 6.6 | 7.4 | 366 | 5.6 | 5.3 | 5.9 | **No** |
|  | **Misuse of pronouns (such as "I" instead of "you")** | **Negative** | 203 | 5.7 | 5.3 | 6.2 | 166 | 5.2 | 4.7 | 5.8 | 316 | 4.8 | 4.4 | 5.03 | **No** |
|  | **Use of unnatural sounds (e.g. high pitch squeal)** | **Negative** | 212 | 7.04 | 6.7 | 7.4 | 170 | 6.02 | 5.6 | 6.5 | 334 | 5.8 | 5.5 | 6.06 | **No** |
|  | **Response to name** | **Positive** | 233 | 5.9 | 5.5 | 6.2 | 151 | 5.4 | 4.9 | 5.8 | 301 | 4.7 | 4.4 | 4.9 | **Negative** |
|  | **Delay in response (social/motor/interactive)** | **Negative** | 233 | 6.1 | 5.8 | 6.4 | 201 | 5.5 | 5.06 | 5.9 | 306 | 4.1 | 3.8 | 4.5 | **No** |
|  | **Avoids eye contact** | **Negative** | 213 | 5.6 | 5.3 | 5.9 | 162 | 5.7 | 5.3 | 6.02 | 320 | 5.5 | 5.3 | 5.8 | **Negative** |
|  | **Understands personal care routine** | **Positive** | 226 | 6.1 | 5.8 | 6.4 | 158 | 5.2 | 4.9 | 5.5 | 358 | 4.8 | 4.6 | 5.04 | **Negative** |
|  | **Fails to express basic needs (e.g. hunger)** | **Negative** | 229 | 4.9 | 4.6 | 5.3 | 187 | 4.2 | 3.8 | 4.5 | 348 | 4.3 | 3.9 | 4.5 | **Positive** |
| **Social Interaction** | **Can s/he start social interactions?** | **Positive** | 235 | 5.8 | 5.4 | 6.1 | 190 | 5.1 | 4.6 | 5.6 | 370 | 5.4 | 5.04 | 5.7 | **Negative** |
|  | **Can s/he maintain social interactions?** | **Positive** | 223 | 5.7 | 5.4 | 6.1 | 166 | 5.0 | 4.6 | 5.4 | 392 | 4.9 | 4.6 | 5.1 | **Negative** |
|  | **Use of social smile** | **Positive** | 97 | 6.04 | 5.4 | 6.7 | 120 | 4.8 | 4.2 | 5.5 | 167 | 3.4 | 2.9 | 3.9 | **Negative** |
| **Problematic Behavior** | **Mood swings** | **Negative** | 172 | 9.09 | 8.9 | 9.2 | 137 | 8.5 | 8.2 | 8.8 | 349 | 6.7 | 6.3 | 7.03 | **No** |
|  | **Self-injurious behavior (frequency)** | **Negative** | 204 | 6.1 | 5.7 | 6.6 | 173 | 2.9 | 2.5 | 3.4 | 349 | 3.9 | 3.6 | 4.2 | **Positive** |
|  | **Self-injurious behavior (intensity)** | **Negative** | 98 | 5.9 | 5.08 | 6.8 | 80 | 4.9 | 3.8 | 5.9 | 119 | 5.09 | 4.3 | 5.9 | **Positive** |
|  | **Aggressive behavior**  **(frequency)** | **Negative** | 130 | 6.7 | 6.3 | 7.08 | 128 | 5.9 | 5.6 | 6.2 | 168 | 5.2 | 4.9 | 5.5 | **No** |
|  | **Aggressive behavior (how often)** | **Negative** | 26 | 2.04 | 1.09 | 2.9 | 30 | 4.7 | 3.7 | 5.7 | 49 | 8.2 | 7.9 | 8.6 | **Negative** |
|  | **Intense interest in objects/parts of objects** | **Positive** | 256 | 6.6 | 6.2 | 6.9 | 177 | 6.07 | 5.7 | 6.5 | 361 | 5.5 | 5.2 | 5.7 | **Negative** |
|  | **Inflexible to change** | **Negative** | 181 | 6.04 | 5.6 | 6.4 | 138 | 5.3 | 4.9 | 5.8 | 294 | 5.9 | 5.6 | 6.3 | **Positive** |
|  | **Repetitive activities (e.g., spinning objects)** | **Negative** | 180 | 8.1 | 7.8 | 8.5 | 155 | 6.9 | 6.5 | 7.3 | 274 | 6.6 | 6.2 | 6.9 | **No** |
|  | **Hyperactive** | **Negative** | 171 | 7.2 | 7.0 | 7.5 | 128 | 7.5 | 7.2 | 7.8 | 226 | 7.3 | 7.1 | 7.5 | **Negative** |
|  | **Lack of concentration** | **Negative** | 175 | 8.06 | 7.9 | 8.4 | 127 | 8.2 | 7.8 | 8.4 | 280 | 7.4 | 7.2 | 7.6 | **Negative** |
| **Sensory Sensitivities** | **Difficulty tracking moving objects/people** | **Negative** | 216 | 6.4 | 5.9 | 6.9 | 187 | 6.1 | 5.7 | 6.6 | 419 | 4.9 | 4.6 | 5.3 | **No** |
|  | **Participation in imaginative games** | **Positive** | 210 | 5.7 | 5.3 | 6.1 | 168 | 5.5 | 5.3 | 6.06 | 303 | 5.2 | 4.9 | 5.4 | **Negative** |
|  | **Sleep problems** | **Negative** | 127 | 5.6 | 5.4 | 6.3 | 146 | 5.8 | 4.9 | 5.9 | 179 | 4.4 | 3.8 | 4.9 | **Negative** |
|  | **Unusual sensitivity to light** | **Negative** | 85 | 3.3 | 2.8 | 3.9 | 101 | 2.08 | 1.7 | 2.5 | 168 | 2.1 | 1.8 | 2.4 | **Positive** |
|  | **Sensitivity to pain** | **Negative** | 56 | 4.6 | 4.3 | 5.5 | 101 | 4.9 | 4.6 | 5.3 | 136 | 5.1 | 4.7 | 5.5 | **Negative** |
|  | **Sensitivity to sound** | **Negative** | 89 | 7.06 | 6.6 | 7.6 | 144 | 5.9 | 5.4 | 6.5 | 349 | 4.9 | 4.5 | 5.2 | **No** |
|  | **Aversion to smell** | **Negative** | 121 | 7.07 | 6.6 | 7.5 | 173 | 5.7 | 5.3 | 6.2 | 348 | 3.9 | 3.6 | 4.3 | **No** |
|  | **Sensitivity to touch** | **Negative** | 52 | 4.7 | 3.9 | 5.5 | 94 | 3.7 | 3.1 | 4.3 | 127 | 3.4 | 2.9 | 3.9 | **No** |
